# Supplementary material for: Modelling Skylarks (Alauda arvensis) to Predict Impacts of Changes in Land Management and Policy: Development and Testing of an Agent-Based Model
Source: PLoS One. 2013 Jun 6;8(6):e65803. doi: 10.1371/journal.pone.0065803 (PMC3675089; doi:10.1371/journal.pone.0065803)
Supplement: Supporting Information S4 — The skylark ODdox as a zipped archive. (ZIP) [file pone.0065803.s004.zip › Skylark_ODdox/classsk_t_territory.html]

ALMaSS Skylark ODdox: skTTerritory Class Reference


ALMaSS Skylark ODdox
 2.0


- Main Page
- Related Pages
- Classes
- Files

- Class List
- Class Index
- Class Hierarchy
- Class Members

Public Member Functions |
Public Attributes |
Protected Attributes

skTTerritory Class Reference

`#include <skylarks_all.h>`

List of all members.

|  |  |
| --- | --- |
| Public Member Functions | |
| int | GetQuality (void) |
| double | GetVirtualDiameter () |
| void | SetNestPossibility (bool valid, int nx, int ny) |
| void | SetQuality (int a\_qual) |
| void | SetVirtualDiameter (double dia) |
|  | skTTerritory (int x, int y, int TheSize, int TheQuality, int a\_x\_div10, int a\_y\_div10, int a\_range\_div10) |
| void | TestNestPossibility () |

|  |  |
| --- | --- |
| Public Attributes | |
| Skylark\_Female \* | F\_Owner |
| double | m\_competitionscaler |
| vector< int > | m\_hr\_polys |
| vector< int > | m\_hr\_sizes |
| int | m\_Location\_x |
| int | m\_Location\_y |
| int | m\_nest\_pos\_validx |
| int | m\_nest\_pos\_validy |
| bool | m\_nest\_valid |
| vector< int > | m\_polys |
| int | m\_range\_div10 |
| vector< int > | m\_sizes |
| int | m\_x\_div10 |
| int | m\_y\_div10 |
| Skylark\_Male \* | Owner |
| int | Size |

|  |  |
| --- | --- |
| Protected Attributes | |
| double | m\_heterogeneity |
| int | m\_Quality |
| double | m\_VirtualDiameter |

---

## Constructor & Destructor Documentation

|  |  |  |  |
| --- | --- | --- | --- |
| skTTerritory::skTTerritory | ( | int | *x*, |
|  |  | int | *y*, |
|  |  | int | *TheSize*, |
|  |  | int | *TheQuality*, |
|  |  | int | *a\_x\_div10*, |
|  |  | int | *a\_y\_div10*, |
|  |  | int | *a\_range\_div10* |
|  | ) |  |  |

References F\_Owner, m\_competitionscaler, m\_heterogeneity, m\_Location\_x, m\_Location\_y, m\_nest\_pos\_validx, m\_nest\_pos\_validy, m\_nest\_valid, m\_Quality, m\_range\_div10, m\_VirtualDiameter, m\_x\_div10, m\_y\_div10, Owner, and Size.

{

m\_x\_div10 = a\_x\_div10;

m\_y\_div10 = a\_y\_div10;

m\_range\_div10 = a\_range\_div10;

m\_Location\_x = x;

m\_Location\_y = y;

Size = TheSize;

m\_VirtualDiameter = Size;

m\_Quality = TheQuality;

Owner = NULL;

F\_Owner = NULL;

m\_competitionscaler = 1.0;

m\_heterogeneity = 1.0;

m\_nest\_valid=false;

m\_nest\_pos\_validx=x;

m\_nest\_pos\_validy=y;

}

---

## Member Function Documentation

|  |  |  |  |  |  |
| --- | --- | --- | --- | --- | --- |
| int skTTerritory::GetQuality | ( | void |  | ) |  |

inline

Referenced by Skylark\_Male::OnMateLeaving(), Skylark\_Male::ReEvaluateTerritory(), and SkTerritories::Supply\_quality().

{ return m\_Quality; }

|  |  |  |  |  |
| --- | --- | --- | --- | --- |
| double skTTerritory::GetVirtualDiameter | ( |  | ) |  |

inline

Referenced by Skylark\_Male::ReEvaluateTerritory(), and SkTerritories::Split().

{ return m\_VirtualDiameter; }

|  |  |  |  |
| --- | --- | --- | --- |
| void skTTerritory::SetNestPossibility | ( | bool | *valid*, |
|  |  | int | *nx*, |
|  |  | int | *ny* |
|  | ) |  |  |

inline

Referenced by SkTerritories::Split().

{

m\_nest\_valid=valid;

m\_nest\_pos\_validx=nx;

m\_nest\_pos\_validy=ny;

}

|  |  |  |  |  |  |
| --- | --- | --- | --- | --- | --- |
| void skTTerritory::SetQuality | ( | int | *a\_qual* | ) |  |

inline

Referenced by SkTerritories::EvaluateHabitatN().

{ m\_Quality = a\_qual; }

|  |  |  |  |  |  |
| --- | --- | --- | --- | --- | --- |
| void skTTerritory::SetVirtualDiameter | ( | double | *dia* | ) |  |

inline

Referenced by SkTerritories::Split().

{ m\_VirtualDiameter = dia; }

|  |  |  |  |  |
| --- | --- | --- | --- | --- |
| void skTTerritory::TestNestPossibility | ( |  | ) |  |

References m\_Location\_x, m\_Location\_y, m\_nest\_pos\_validx, m\_nest\_pos\_validy, m\_nest\_valid, m\_Quality, m\_range\_div10, Landscape::SupplyElementType(), Landscape::SupplySimAreaWidth(), tole\_ActivePit, tole\_Building, tole\_ConiferousForest, tole\_DeciduousForest, tole\_Garden, tole\_Hedges, tole\_MixedForest, tole\_RiversideTrees, tole\_StoneWall, and tole\_Urban.

Referenced by SkTerritories::PreProcessLandscape2().

{

bool result=false;

bool\* terrsurface = new bool [m\_range\_div10\*m\_range\_div10];

for (int i=0; i<m\_range\_div10; i++) {

for (int j=0; j<m\_range\_div10; j++) {

terrsurface[i+j\*m\_range\_div10]=true;

}

}

int skAvoid = \_\_SKTALLAVOIDENCE/10;

for (int rx=-skAvoid; rx<m\_range\_div10+skAvoid; rx++) {

int rrx=rx\*10;

for (int ry=-skAvoid; ry<m\_range\_div10+skAvoid; ry++) {

int rry=ry\*10;

for (int sx=0; sx<10; sx++) {

for (int sy=0; sy<10; sy++) {

int x = m\_Location\_x+sx+rrx;

int y = m\_Location\_y+sy+rry;

if ((x<0) || (y<0) || (x>=g\_land->SupplySimAreaWidth()) || (y>=g\_land->SupplySimAreaWidth())) {

;

}

else {

TTypesOfLandscapeElement ele = g\_land->SupplyElementType(x,y);

switch (ele) {

case tole\_Hedges:

//case tole\_HedgeBank: // \*\*\*CJT\*\*\* testing with and without this, h desigination to exe

case tole\_RiversideTrees: // 97

case tole\_DeciduousForest: // 40

case tole\_MixedForest: // 60

case tole\_ConiferousForest: // 50

case tole\_StoneWall: // 15

case tole\_Building: // 5

case tole\_Urban: // 10

case tole\_Garden: // 10

case tole\_ActivePit: // 115

result=true;

// No point in testing the rest of this square

sx=11;

sy=11;

break;

default:

;

}

}

}

}

if (result) {

// Get the location in our 10x10m grid

int gx = rx;

int gy = ry;

// Invalidate the area around this point

int tx1 = gx+skAvoid;

int tx2 = gx-skAvoid;

int ty1 = gy+skAvoid;

int ty2 = gy-skAvoid;

if (tx1>m\_range\_div10) tx1=m\_range\_div10;

if (ty1>m\_range\_div10) ty1=m\_range\_div10;

if (tx2<0) tx2=0;

if (ty2<0) ty2=0;

for (int xx=tx2; xx<tx1; xx++) {

for (int yy=ty2; yy<ty1; yy++) {

terrsurface[xx+yy\*m\_range\_div10]=false;

}

}

}

result=false;

}

}

m\_nest\_valid = false;

for (int i=0; i<m\_range\_div10; i++) {

for (int j=0; j<m\_range\_div10; j++) {

if (terrsurface[i+j\*m\_range\_div10]) {

m\_nest\_valid=true;

m\_nest\_pos\_validx = m\_Location\_x + (10\*i);

m\_nest\_pos\_validy = m\_Location\_y + (10\*j);

i=m\_range\_div10;

break;

}

}

}

delete [] terrsurface;

if (!m\_nest\_valid) m\_Quality=0;

}

---

## Member Data Documentation

Skylark\_Female\* skTTerritory::F\_Owner

Referenced by SkTerritories::FemaleOccupy(), SkTerritories::RemoveFemale(), skTTerritory(), and SkTerritories::Supply\_F\_Owner().

double skTTerritory::m\_competitionscaler

Referenced by SkTerritories::EvaluateHabitatN(), skTTerritory(), and SkTerritories::Split().

double skTTerritory::m\_heterogeneity

protected

Referenced by skTTerritory().

vector< int > skTTerritory::m\_hr\_polys

Referenced by Skylark\_Male::ConstructAHabitatTable(), and SkTerritories::PreFillTerrPolyLists().

vector< int > skTTerritory::m\_hr\_sizes

Referenced by Skylark\_Male::ConstructAHabitatTable(), and SkTerritories::PreFillTerrPolyLists().

int skTTerritory::m\_Location\_x

Referenced by SkTerritories::DumpMapGraphics(), skTTerritory(), SkTerritories::Supply\_x(), and TestNestPossibility().

int skTTerritory::m\_Location\_y

Referenced by SkTerritories::DumpMapGraphics(), skTTerritory(), SkTerritories::Supply\_y(), and TestNestPossibility().

int skTTerritory::m\_nest\_pos\_validx

Referenced by skTTerritory(), and TestNestPossibility().

int skTTerritory::m\_nest\_pos\_validy

Referenced by skTTerritory(), and TestNestPossibility().

bool skTTerritory::m\_nest\_valid

Referenced by skTTerritory(), and TestNestPossibility().

vector< int > skTTerritory::m\_polys

Referenced by SkTerritories::EvaluateHabitatN(), and SkTerritories::PreFillTerrPolyLists().

int skTTerritory::m\_Quality

protected

Referenced by skTTerritory(), and TestNestPossibility().

int skTTerritory::m\_range\_div10

Referenced by SkTerritories::PreFillTerrPolyLists(), skTTerritory(), and TestNestPossibility().

vector< int > skTTerritory::m\_sizes

Referenced by SkTerritories::EvaluateHabitatN(), and SkTerritories::PreFillTerrPolyLists().

double skTTerritory::m\_VirtualDiameter

protected

Referenced by skTTerritory().

int skTTerritory::m\_x\_div10

Referenced by SkTerritories::PreFillTerrPolyLists(), and skTTerritory().

int skTTerritory::m\_y\_div10

Referenced by SkTerritories::PreFillTerrPolyLists(), and skTTerritory().

Skylark\_Male\* skTTerritory::Owner

Referenced by SkTerritories::Occupy(), SkTerritories::RemoveMale(), skTTerritory(), and SkTerritories::Supply\_Owner().

int skTTerritory::Size

Referenced by SkTerritories::DumpMapGraphics(), skTTerritory(), and SkTerritories::Supply\_size().

---

The documentation for this class was generated from the following files:

- skylarks\_all.h
- skylarks\_all.cpp


- skTTerritory
- Generated on Thu Jan 10 2013 13:15:37 for ALMaSS Skylark ODdox by
   1.8.1.1
